# From market access to patient access: overview of evidence-based approaches for the reimbursement and pricing of pharmaceuticals in 36 European countries

**DOI:** 10.1186/s12961-015-0028-5

**Published:** 2015-09-25

**Authors:** Dimitra Panteli, Helene Eckhardt, Alexandra Nolting, Reinhard Busse, Michael Kulig

**Affiliations:** Department of Health Care Management, Berlin University of Technology, Berlin, Germany; Federal Joint Committee (G-BA), Berlin, Germany

## Abstract

**Background:**

Coverage decisions determining the benefit baskets of health systems have been increasingly relying on evidence regarding patient benefit and costs. Relevant structures, methodologies, and processes have especially been established for pharmaceuticals but approaches differ. The objective of this work was thus to identify institutions in a broad range of European countries (n = 36) in charge of determining the value of pharmaceuticals for pricing and reimbursement purposes and to map their decision-making process; to examine the different approaches and consider national and supranational possibilities for best practice.

**Methods:**

Institutions were identified through websites of international networks, ministries, and published literature. Details on institutional practices were supplemented with information from institution websites and linked online sources.

**Results:**

The type and extent of information available varied considerably across countries. Different types of public regulatory bodies are involved in pharmaceutical coverage decisions, assuming a range of responsibilities. As a rule, the assessment of scientific evidence is kept structurally separate from its appraisal. Recommendations on value are uniformly issued by specific committees within or commissioned by responsible institutions; these institutions often also act as decision-makers on reimbursement status and level or market price. While effectiveness and costs are important criteria in all countries, the latter are often considered on a case-by-case basis. In all countries, manufacturer applications, including relevant evidence, are used as one of the main sources of information for the assessment.

**Conclusion:**

Transparency of evidence-based coverage decisions should be enhanced. International collaboration can facilitate knowledge exchange, improve efficiency of information production, and strengthen new or developing systems.

**Electronic supplementary material:**

The online version of this article (doi:10.1186/s12961-015-0028-5) contains supplementary material, which is available to authorized users.

## Background

Coverage decisions for health technologies are increasingly incorporating evidence-based approaches [1,2]. While the post-marketing evaluation of health technologies was initially motivated by goals such as timely access to appropriate new technologies and the efficient allocation of finite resources [1], the move towards a more systematic implementation of evidence-based approaches has been advanced by increasing financial constraints and a general focus on the scientific basis of decision-making in healthcare [1,2].

Most formally established decision structures that rely on evidence concern the reimbursement and pricing of pharmaceuticals [2]. These were introduced as a way to obtain value for money in addition to existing measures of cost control targeting pharmaceutical expenditure. According to the Organisation for Economic Co-operation and Development, outpatient pharmaceutical expenditure alone was the third largest item within total health expenditures among its member countries in 2011 [3]. In the European Union (EU), cost-containment measures include external and internal reference pricing, regulatory steering of market access, public tendering, price freezes, cuts and discounts, regulation of profit margins for pharmacists and wholesalers, patient cost-sharing, guidelines for and monitoring of prescribing behaviours, and generic substitution [4,5].

Post-marketing evaluations of pharmaceuticals can vary with regard to scope and methodology depending on their exact purpose and the health system in which they take place [6,7]. In Europe, health technology assessment has been increasingly implemented in this context since the beginning of the 2000s [5]. For the purpose of this paper, we use the term ‘health technology assessment’ (HTA) to describe scientifically sound and transparently produced evidence syntheses which cover a variable range of evidence domains and aim to inform coverage decision-makers; we understand ‘relative effectiveness’ as “*the extent to which an intervention does more good than harm compared with one or more intervention alternatives for achieving the desired results when provided under the usual circumstances of healthcare practice*” [1].

Pharmaceutical pricing and reimbursement modalities in different countries have been the subject of an increasing volume of comparative research in recent years (an overview of relevant comparative publications can be found in Additional file 1). While work on the underlying decision-making processes has been variable in regional and thematic focus as well as methodological approach, it has consistently found that evidence requirements [8-11], as well as the characteristics of the evaluation process [12,13], show similarities but also important differences across contexts; the same is true for the number of stakeholders involved in evidence-based coverage decisions and their interactions [14-16].

An overarching goal of the aforementioned research has been the consideration and evaluation of current approaches towards evidence-based coverage decisions to inform best practice. Recognizing that the analysis of coverage decision-making is complicated in nature [17] and requires, as a first step, a clear identification of stakeholders involved, this paper aims to build on previous insights and provide decision-makers and researchers with an updated, concise overview of processes and responsibilities in a broad range of European countries (n = 36). Furthermore, it aims to illustrate differing approaches and consider national and supranational possibilities for best practice. As a secondary objective, the paper also set out to assess the extent to which publicly available information on these issues sufficiently allows for a transparent view on decision-making.

## Methods

### Rationale

The landscape of coverage decision-making encompasses a multitude of stakeholders and dynamics [17]. This work focused on active regulatory institutions charged with the evaluation of and/or decision-making on the value of pharmaceuticals for public reimbursement or pricing in EU Member States, candidate countries, and the European Free Trade Association countries. Processes at regional level were not specifically targeted in countries with decentralized systems, with the exception of the United Kingdom, where the systems for England and Wales, and Scotland were studied separately. While the exact definition of value may vary across systems and countries, benefit (in terms of patient health gain) is the main element of interest, while costs (in terms of lower out-of-pocket payments or improved efficiency in the system) can be another key consideration [18,19].

**Figure 1:**
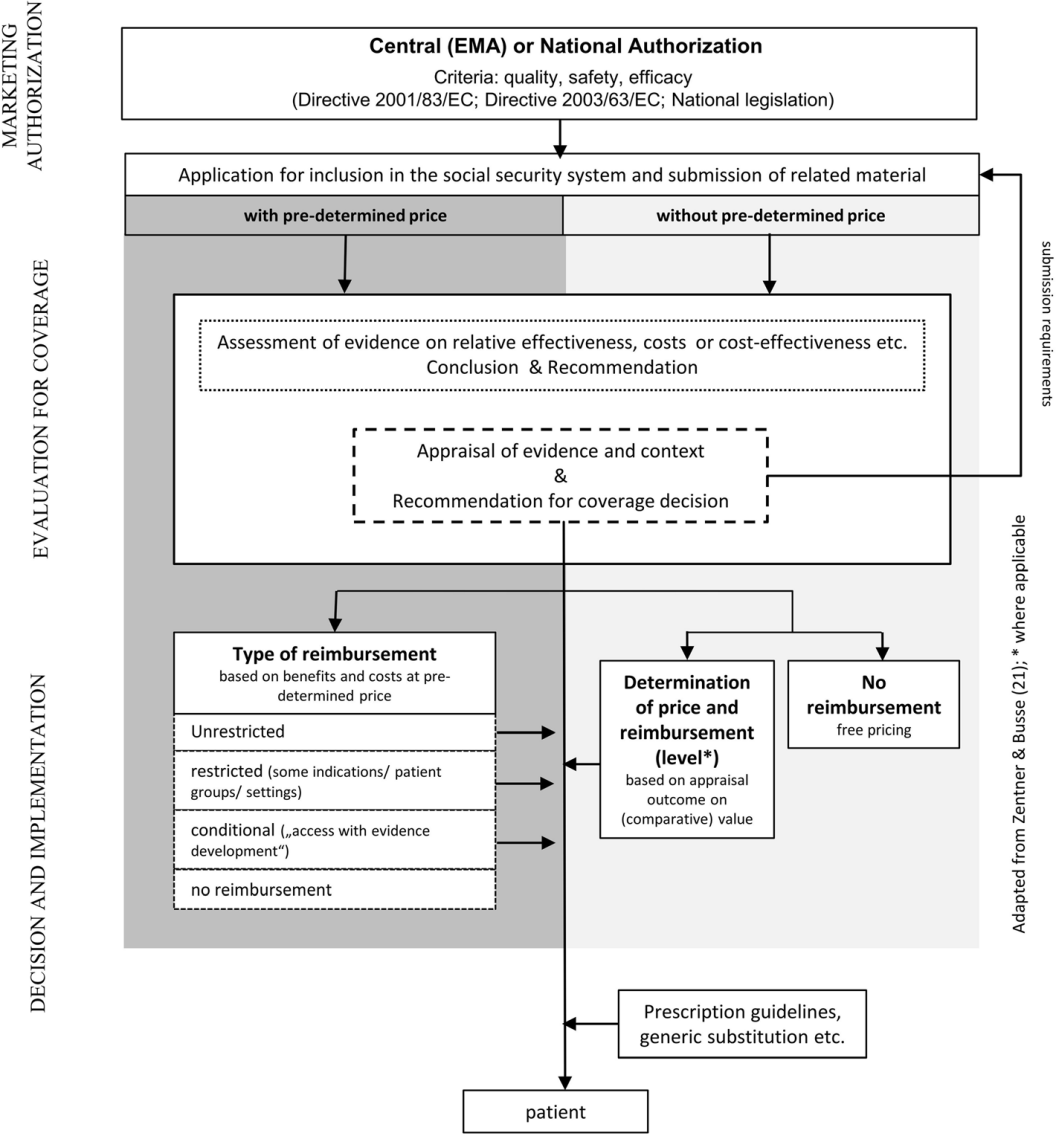
**Evaluation of newly authorized pharmaceuticals for the purpose of reimbursement and pricing**

We used the analytical frameworks proposed by Hutton et al. [2] and Rogowski et al. [20] to develop an information collection tool tailored to our research objectives (Table 1). Hutton et al. [2] proposed a two-level framework, distinguishing between the ‘policy implementation level’ of post-licensing evaluations overall and the ‘individual technology decision level’, which examines decision-making processes for individual technologies. Rogowski et al. [20] allow for seven building blocks in the analysis of coverage decisions (trigger, participation, assessment, appraisal, reimbursement, publication, management). The concept behind our work is illustrated by the shaded area in Figure 1. Figure 1 provides a simplified depiction of decision-making pathways for pharmaceuticals from marketing authorization to patient access, distinguishing between countries where pharmaceuticals are evaluated for reimbursement based on a price set in advance and countries where price and reimbursement are determined once the evaluation has been completed [21] (see also a similar distinction by Towse and Barnsley [18]).

**Table 1:** Elements of information extraction tool on country processes

Decision-making on the value of pharmaceuticals	
• Responsible Institution/Body	
• Application area (remit)	
• Evidence assessment	
✠ production of evidence report	
✠ assessment of evidence report	
✠ assessment staff	
• Evidence appraisal	
✠ voting body and members	
✠ term of office	
• Re-Assessment	
✠ Regular versus ad hoc	
✠ Initiation (who/why)	
Decision making on reimbursement and pricing of pharmaceuticals	
• Responsible Institution / Body	
• Restrictions

### Data collection

Our approach followed three distinct steps: (1) relevant institutions were identified for each country, (2) information was extracted from online sources, and (3) collected information was sent to the identified institutions for validation.

To identify relevant institutions, we searched for information on the websites of international organisations (European Network for Health Technology Assessment (EUnetHTA), European Observatory on Health Systems and Policies, International Society for Pharmacoeconomics and Outcomes Research (ISPOR), WHO Collaborating Centre for Pharmaceutical Pricing and Reimbursement) and all related national ministries. Information was supplemented by a PubMed search for relevant literature (see Additional file 2 for search strategy) which served as a cross-check to ensure that information was complete and well-founded.

Once the relevant institutions in each country had been identified, information on processes and institutional requirements related to evidence-based decision making was collected using online sources. Institutional websites were searched in a structured manner using the site map and search functions (where available) as well as linked material. Google Translate was used to extract information from websites where no information was available in one of the working languages (English, French, German, Greek, and Russian). The plausibility of resulting interpretations was checked by two investigators in each case. Synthesised information on each country was used to create individual country profiles. The structured search for information took place in summer 2012 and was repeated in February 2015 to ensure validity of information. Additional file 3 provides a list of visited links per country.

After the initial search, all institutions included in the analysis were contacted through email to verify the output using the contact information provided by each website (with up to five periodic reminders for non-respondents).

## Results

An overview of information on all included countries corresponding to the elements in Table 1 is presented in Additional file 4: Table S1. Bolded country names correspond to those cases where Google Translate had to be used to extract information.

### Institutions included in analysis and information availability

Institutions in charge of decision-making on reimbursement and pricing of pharmaceuticals were identified in 34 out of 36 countries included in the initial pool. In Liechtenstein and Macedonia, there is no separate institution tasked with these decisions. Liechtenstein generally adopts Switzerland’s positive list (Arznei- und Spezialitätenliste) and in Macedonia ad hoc committees are summoned by the Ministry of Health.

The type and extent of information available both on the evaluation of relative effectiveness and on decision-making for pharmaceutical coverage varies. For instance, information is generally sparse (e.g. Greece, Luxembourg) or incomplete (e.g. Czech Republic, Ireland, Iceland, Norway, Poland, Turkey). Unfortunately, despite repeated attempts to obtain verification of the information by country experts, few responses were forthcoming (Czech Republic, Hungary, Liechtenstein, Malta, Norway, Republic of Serbia); consequently, no contact was attempted after the update search in February 2015. In most cases, contacted institutions provided additional details or suggested rephrasing; one institution confirmed that information was complete and accurate (Liechtenstein).

### Decision-makers responsible for pharmaceutical coverage

Central processes to determine whether pharmaceuticals are to be reimbursed and to set their price are initiated after marketing authorization has been granted. Different public regulatory bodies are involved across Europe, including Ministries of Health/Social Affairs, Social Health Insurance Organisations, National Health Service Executives, or Medicines Agencies.

In some countries, the same agencies are also in charge of pharmaceutical marketing authorisation (e.g. Denmark, Greece, Iceland, Italy). Similarly, while in many countries the same regulatory institutions are responsible for the final political decision on a pharmaceutical’s price and reimbursement status, in others, final approval by a different instance is required (e.g. Belgium, France, United Kingdom, Malta, the Netherlands, Portugal, Republic of Serbia, Turkey).

Special groups within or independent public bodies commissioned by the aforementioned institutions are responsible for the assessment of the pharmaceutical’s relative effectiveness. These committees or institutions have been established in almost all European countries included in the analysis. In Greece, pharmaceuticals are included in the positive list, if they have undergone affirmative assessment in other EU countries. The positive list was reinstated in 2011 amidst the financial crisis.

### Remit of coverage decision-makers

Evaluation is mostly carried out for newly authorised pharmaceuticals with new substances either exclusively for the out-patient sector (n = 11) or for both the out- and in-patient sectors. In a range of countries, already reimbursed medicines are reassessed (see related section below). Approximately half of the institutions studied are also directly involved in price determination.

### The assessment process

Assessments of the (relative) effectiveness of pharmaceuticals are performed either by a separate committee within the institution itself or a commissioned institution. In all countries, manufacturers are required to submit evidence-based applications, which are used as one of the main sources of information for the assessment. In specific cases, the evaluation committees or the commissioned institutions (additionally) produce evidence reports themselves (e.g. France, Sweden, United Kingdom–England and Wales). Assessment groups consist of five to thirty members, comprising scientists from different disciplines including pharmacologists, pharmacists, epidemiologists, physicians, economists, mathematicians, and statisticians.

The criteria underpinning the evaluation of pharmaceuticals for reimbursement and pricing decisions are based on national legislation. One important criterion in all countries is the assessment of (relative) effectiveness. Costs also seem to play a significant role in all countries where information on the issue was available (see Additional file 4: Table S1), although they are often (n = 15) considered on a case-by-case basis.

As a rule, the assessment staff receives submissions from manufacturers, assesses the submitted material based on clinical and/or economic criteria, completes or produces evidence reports where required, and finally composes conclusions or recommendations on the pharmaceutical’s relative effectiveness. However, evidence requirements and the degree of public availability of submitted material vary from country to country.

### The appraisal process

European countries keep the process of assessment of scientific evidence structurally separate from its appraisal and from final decision-making on reimbursement status, reimbursement level, or price. Reimbursement or pricing committees or institutions discuss the results of the completed reports provided by assessment groups in context and formulate conclusions and give advice to the final decision-makers or act as final decision-makers themselves (see Additional file 4: Table S1).

In most cases, assessment groups and reimbursement/pricing committees represent the same body but differ in composition. While assessment staff commonly consists of scientists, a reimbursement/pricing committee additionally includes representatives of ministries, healthcare provider organisations (e.g. physicians and pharmacists), health insurers, and representatives of patient groups. In some countries, representatives of the pharmaceutical industry are also involved. However, their role in the process varies: while in Belgium, France, Romania, and England and Wales industry representatives act only as non-voting participants in the committee, in other countries (e.g. Switzerland, Turkey, United Kingdom–Scotland) they are full members. The committees may additionally consult external experts.

The terms of office of pricing/reimbursement committee members range from 1 to 8 years. Discussion and appraisal usually take place in closed sessions but the minutes of these sessions are made publicly available in the majority of countries for which information was available (except in Estonia, Spain, Macedonia, Montenegro, Portugal, Turkey).

### Outcomes of appraisal and reimbursement restrictions

Reimbursement levels can depend on product specifications, on the medical condition (indication), or on the patient or population group. There can also be limitations on the quantity of prescribed medicines, or restrictions regarding prescribers, on specific physicians and/or medical institutions or treatment.

### Re-evaluation of pharmaceuticals

Reviews of previous reimbursement or pricing decisions can be substance-specific and occur on request of manufacturers or by initiative of the reimbursement/pricing institution or commission. They can be triggered by new pharmacological, medical-therapeutic, or health-economic evidence, or because of changes in indication or price (Additional file 4: Table S1). Re-evaluations can also be initiated by ministries or at the request of representatives of interested parties such as patients, third party payers, physicians, scientists, etc. In some countries (e.g. Belgium, Finland), a regular review of coverage decisions on new pharmaceuticals is scheduled every 1–5 years. In others, the entire range of reimbursed pharmaceuticals is subject to a regular review every 4–5 years (e.g. Spain, the Netherlands).

### Transparency of process of assessment and decision-making

The structured search identified gaps in information on several dimensions of the assessment and decision-making process for reimbursement and pricing of pharmaceuticals in many of the 36 countries analysed, as indicated by missing information in Additional file 4: Table S1. Moreover, available information often required considerable effort to identify and was sometimes only included in legal acts or regulatory documents. This was interpreted as a general lack of accessibility to information and therefore transparency in many cases.

## Discussion

Our findings confirm previous research indicating that evidence-based decision-making processes for reimbursement and pricing of pharmaceuticals in Europe demonstrate a number of similarities even in fundamentally different healthcare systems. Thus, manufacturer applications including relevant scientific evidence are always required for a pharmaceutical to be (re)considered for coverage and build the basis of the evidence report guiding decisions. The assessment and appraisal of the evidence is kept structurally separate, even when carried out within the same institution. Both assessment and appraisal groups are multidisciplinary in nature but differ in constitution: the former include primarily scientists and methods experts while the latter tend to incorporate a multitude of stakeholders such as third party payers and occasionally patient or industry representatives (for more detail on commission composition see also [16]).

Despite the similarities discussed above, no two systems are identical in terms of procedure or content of evidence considered. This has also been discussed by Allen et al. [15], who systematized HTA-related processes in Europe into 10 archetypes and found that the set-up of HTA systems was not connected to a country’s geographical location or ability to pay. Taking into account our findings on the variability of HTA processes and the lack of apparent clustering, one could assume - as Allen et al. [15] did - that the historical development of the legal and regulatory framework of the health system as well political aspects can lead to the differences observed in evidence-based decision-making processes. Furthermore, it is likely that elements from country models already in place informed the development of newer systems, thus contributing to observed similarities.

The assessment of scientific evidence is kept separate from contextualized appraisals and has been found to show important similarities across European countries [9]. Collaboration platforms exist both at European (EUnetHTA) and international levels (International Network of Agencies for Health Technology Assessment (INAHTA), Health Technology Assessment International (HTAi)). The collaboration of institutions involved in HTA at the national level has further been endorsed by the European Commission in its Directive on the application of patients’ rights in cross-border healthcare, to be facilitated by EUnetHTA (Directive 2011/24/EU). The Directive recognizes the importance of information exchange in providing safe and appropriate technologies for patients in an overarching way. One of the main aims of EUnetHTA since its inception in 2007 has been to facilitate the prevention of duplication of efforts [22]. In addition to facilitating best practice, information exchange may also be cost-effective in its own right [23].

Coverage decisions, on the other hand, are taken at national (or regional) level. Perhaps as contributing factors to this fact, substantial differences in the organization and processes of decision-making institutions as well as in the spectrum of possible decision outcomes can be observed. However, communication platforms and the possibility for knowledge exchange can be important for countries where pathways for evidence-based decision-making are still being developed or difficult to introduce. While HTA may be a uniquely appropriate approach for an efficient allocation of resources in constrained systems, the necessary legal and organizational structures are not easy to establish [24]. Greece, for example, only introduced HTA as a criterion for coverage in light of the financial crisis and only considers pharmaceuticals for reimbursement if they have been favourably evaluated in other countries [25]. Liechtenstein has traditionally been adopting decisions made in the Swiss system. It could therefore be beneficial for appraisal committees to have a platform enabling structured dialogue, for example on the validity, appropriateness and adaptability of evidence reports produced for different contexts. Such a platform could furthermore facilitate discussion on the implementation of reimbursement restrictions, which differ across countries in terms of both rationale and frequency of application [26].

The lack of transparency observed in many cases may pose an obstacle to cross-national collaboration. One of the aims of this work was to explore publicly available information which was found to be fragmented or lacking and difficult to obtain for many countries and for several elements of the process such as the appointment and term of appraisal committee members or the process of re-evaluation. This has also been observed in previous comparative research [12,14,16]. While the impact of transparency on the reasonableness of decisions is contested [27] it is clear that it remains paramount in regard to accountability, consistency of implementation, and the right to information of a broad range of stakeholders, not least patients. Given the role of manufacturer submissions outlined above, an important point to consider at this juncture is that of ‘commercial in confidence’ information. The extent to which such data is accepted by regulatory institutions varies [28]. In the interest of transparency, institutional policies on the consideration of confidential information should be clearly stated.

Stakeholder participation and thorough evidence assessment have been found to be of particular importance for the scientific rigor and reasonableness of coverage decisions [29]. Stakeholder participation is particularly diverse across systems and an overall pattern is difficult to distinguish. While most countries included in our study provide explicit requirements on which manufacturer submissions should be based, these vary in terms of content and comprehensiveness. Despite the similarities observed in scientific approaches within coverage decision-making systems [9], there seems to be a wide variation in the thoroughness of process for evidence assessment and appraisal. Building on previous initiatives such as the HTAi Policy Forum [1], international dialogue among expert stakeholders can contribute to overcoming methodological or procedural challenges.

Our work is not without limitations. As per the research objectives, online sources were used to obtain information. Thus, any facet of the process not illustrated in these sources was left out. Furthermore, the use of Google Translate comes with its own validity constraints. While all efforts were made to check rendered content, the possibility of misinterpretation cannot be unequivocally excluded. Finally, normative statements made in the available documentation were taken at face value and were not compared to actual cases of coverage decision-making.

The focus of this work was on the reimbursement and pricing of pharmaceuticals. However, many countries are expanding the range of technologies undergoing evidence-based assessments to include medical devices, procedures, and public health interventions. It is important to note that reimbursement and pricing systems for other technologies, such as medical devices, differ considerably from those described here [30]. Evidence-based decisions for such technologies can thus be additionally complicated by contextual factors such as less confluent marketing authorization processes or concrete definition systems. Finally, the type and composition of actors with final decision-making power in matters of coverage and the criteria they employ have not been explored in a comprehensive way and were not covered in detail in our work. Future comparative analyses could aim to expand existing knowledge and bring clarity to the issue.

## Conclusions

Different public regulatory bodies are involved in pharmaceutical coverage decisions across Europe. The assessment and appraisal of evidence is kept structurally separate and based on material submitted by manufacturers. Reassessment policies and reimbursement restrictions vary considerably and transparency of process is limited in many cases. International collaboration among HTA and regulatory bodies can promote knowledge exchange and efficiency of information production. It can thus help refine existing approaches and improve evidence-based coverage decision-making as well as prepare developing systems for new challenges.

## Additional files

Additional file 1:
**Overview of comparative peer-reviewed publications on elements of reimbursement and pricing.** (PDF 29 kb) Additional file 2:
**Search strategy for the PubMed search on institutions responsible for reimbursement and pricing.** (DOCX 19 kb) Additional file 3:
**Sources per country.** (DOCX 34 kb) Additional file 4: Table S1. Overview of evidence-based decision-making processes for pharmaceutical coverage in 36 countries. (DOCX 62 kb)

## Abbreviations

EU: European Union
EUnetHTA: European Network for Health Technology Assessment
HTA: Health Technology Assessment
HTAi: Health Technology Assessment International
INAHTA: International Network of Agencies for Health Technology Assessment
ISPOR: International Society For Pharmacoeconomics and Outcomes Research
