# Supplementary material for: From market access to patient access: overview of evidence-based approaches for the reimbursement and pricing of pharmaceuticals in 36 European countries
Source: Health Res Policy Syst. 2015 Sep 25;13:39. doi: 10.1186/s12961-015-0028-5 (PMC4583728; doi:10.1186/s12961-015-0028-5)
Supplement: Additional file 2: — Search strategy for the PubMed search on institutions responsible for reimbursement and pricing. (DOCX 19 kb) [file 12961_2015_28_MOESM2_ESM.pdf]

Medline (Pubmed), date of search 2012-04-12

#1	Search ((((((pharmaceuticals[TIAB])) OR (drugs[TIAB])) OR (medicines[TIAB])) OR ("Pharmaceutical Preparations"[Mesh]))) AND (((((((reimbursement[TIAB])) OR (pricing[TIAB])) OR ("Insurance Coverage"[Mesh])) OR ("Costs and Cost Analysis"[Mesh])) OR ("Insurance, Health, Reimbursement"[Mesh])) OR ("Insurance Benefits"[Mesh]))	12231
#2	Search portugal[TIAB]	5092
#3	Search portuguese[TIAB]	4747
#4	Search "Portugal"[Mesh]	6073
#5	Search ((#2) OR #3) OR #4	10897
#6	Search (#5) AND #1	20
#7	Select 20 document(s)	20
#8	Search romania*[TIAB]	3201
#9	Search "Romania"[Mesh]	7354
#10	Search (#8) OR #9	8572
#11	Search (#10) AND #1	7
#12	Select 7 document(s)	7
#13	Search slovakia*[TIAB]	2264
#14	Search "Slovakia"[Mesh]	1486
#15	Search (#13) OR #14	2948
#16	Search (#15) AND #1	14
#17	Select 14 document(s)	14
#18	Search slovenia*[TIAB]	2018
#19	Search "Slovenia"[Mesh]	1382
#20	Search (#18) OR #19	2397
#21	Search (#20) AND #1	9
#22	Select 9 document(s)	9
#23	Search spain[TIAB]	28464
#24	Search spanish[TIAB]	22133
#25	Search "Spain"[Mesh]	45852
#26	Search ((#23) OR #24) OR #25	68316
#27	Search (#26) AND #1	265
#28	Select 265 document(s)	265
#29	Search sweden[TIAB]	27124
#30	Search swedish[TIAB]	22201
#31	Search "Sweden"[Mesh]	51637
#32	Search ((#29) OR #30) OR #31	70457
#33	Search (#32) AND #1	284
#34	Select 284 document(s)	284
#35	Search united kingdom[TIAB]	21755
#36	Search great britain[TIAB]	5559
#37	Search england[TIAB]	28581
#38	Search english[TIAB]	42906
#39	Search british[TIAB]	33062
#40	Search "England"[Mesh]	80622
#41	Search "Great Britain"[Mesh]	269425
#42	Search scotland[TIAB]	10361
#43	Search scottish[TIAB]	5569
#44	Search "Scotland"[Mesh]	18835
#45	Search (((((((((#35) OR #36) OR #37) OR #38) OR #39) OR #40) OR #41) OR #42) OR #43) OR #44	355596
#46	Search (#45) AND #1	946
#47	Select 946 document(s)	946

#48	Search croatia*[TIAB]	4662
#49	Search "Croatia"[Mesh]	4088
#50	Search (#48) OR #49	5931
#51	Search (#50) AND #1	19
#52	Select 19 document(s)	19
#53	Search macedonia*[TIAB]	624
#54	Search "Macedonia (Republic)"[Mesh]	218
#55	Search ((#53) OR #54) AND #1	2
#56	Select 2 document(s)	2
#57	Search iceland*[TIAB]	3918
#58	Search "Iceland"[Mesh]	2908
#59	Search (#57) OR #58	4762
#60	Search (#59) AND #1	8
#61	Select 8 document(s)	8
#62	Search montenegro[TIAB]	467
#63	Search "Montenegro"[Mesh]	48
#64	Search (#62) OR #63	482
#65	Search (#64) AND #1	0
#66	Search turkey[TIAB]	19341
#67	Search turkish[TIAB]	8370
#68	Search "Turkey"[Mesh]	18142
#69	Search ((#66) OR #67) OR #68	31859
#70	Search (#69) AND #1	29
#71	Select 29 document(s)	29
#72	Search liechtenstein[TIAB]	170
#73	Search "Liechtenstein"[Mesh]	24
#74	Search (#72) OR #73	175
#75	Search (#74) AND #1	1
#76	Select 1 document(s)	1
#77	Search norway[TIAB]	18277
#78	Search norwegian[TIAB]	10917
#79	Search "Norway"[Mesh]	26667
#80	Search ((#77) OR #78) OR #79	30374
#81	Search (#80) AND #1	89
#82	Select 89 document(s)	89
#83	Search switzerland[TIAB]	13227
#84	Search swiss[TIAB]	20449
#85	Search "Switzerland"[Mesh]	24589
#86	Search ((#83) OR #84) OR #85	46592
#87	Search (#86) AND #1	81
#88	Select 81 document(s)	81
#89	Search austria*[TIAB]	9948
#90	Search "Austria"[Mesh]	14236
#91	Search (#89) OR #90	19632
#92	Search (#91) AND #1	52
#93	Search "Belgium"[Mesh]	12125
#94	Search belgi*[TIAB]	12949
#95	Search (#93) OR #94	19101
#96	Search (#95) AND #1	88
#97	Search bulgaria*[TIAB]	3823
#98	Search "Bulgaria"[Mesh]	5146

#99	Search (#97) OR #98	6758
#100	Search (#99) AND #1	11
#101	Select 11 document(s)	11
#102	Search cyprus[TIAB]	889
#103	Search cypriot*[TIAB]	327
#104	Search (#102) OR #103	1077
#105	Search (#104) AND #1	1
#106	Select 1 document(s)	1
#107	Search czech*[TIAB]	11003
#108	Search "Czech Republic"[Mesh]	4151
#109	Search (#107) OR #108	12410
#110	Search (#109) AND #1	31
#111	Select 31 document(s)	31
#112	Search (denmark[TIAB]) OR danish[TIAB]	26512
#113	Search "Denmark"[Mesh]	33498
#114	Search (#112) OR #113	44883
#115	Search (#114) AND #1	122
#116	Select 122 document(s)	122
#117	Search "Estonia"[Mesh]	1617
#118	Search (estonia*[TIAB]) OR #57	2311
#119	Search (#117) OR #118	44883
#120	Search (#119) AND #1	5
#121	Select 5 document(s)	5
#122	Search finland[TIAB]	15635
#123	Search finish[TIAB]	2839
#124	Search "Finland"[Mesh]	25193
#125	Search ((#122) OR #123) OR #124	28012
#126	Search (#125) AND #1	52
#127	Select 52 document(s)	52
#128	Search france[TIAB]	35892
#129	Search french[TIAB]	37314
#130	Search "France"[Mesh]	69885
#131	Search ((#128) OR #129) OR #130	109032
#132	Search (#131) AND #1	376
#133	Select 376 document(s)	376
#134	Search german*[TIAB]	78658
#135	Search "Germany"[Mesh]	113364
#136	Search (#134) OR #135	158165
#137	Search (#136) AND #1	516
#138	Select 516 document(s)	516
#139	Search greece[TIAB]	8010
#140	Search greek[TIAB]	7278
#141	Search "Greece"[Mesh]	12110
#142	Search ((#140) OR #82) OR #141	18821
#143	Search (#142) AND #1	28
#144	Select 28 document(s)	28
#145	Search hungary[TIAB]	6644
#146	Search hungarian[TIAB]	4531
#147	Search "Hungary"[Mesh]	14629
#148	Search ((#145) OR #146) OR #147	18641
#149	Search (#148) AND #1	36

#150	Select 36 document(s)	36
#151	Search ireland[TIAB]	10882
#152	Search irish[TIAB]	4994
#153	Search "Ireland"[Mesh]	11188
#154	Search ((#151) OR #152) OR #153	13417
#155	Search (#154) AND #1	33
#156	Select 33 document(s)	33
#157	Search italy[TIAB]	33945
#158	Search italian[TIAB]	25642
#159	Search "Italy"[Mesh]	61304
#160	Search ((#157) OR #158) OR #159	86457
#161	Search (#160) AND #1	233
#162	Select 233 document(s)	233
#163	Search latvia*[TIAB]	862
#164	Search "Latvia"[Mesh]	878
#165	Search (#163) OR #164	1257
#166	Search (#165) AND #1	0
#167	Search Lithuania*[TIAB]	1741
#168	Search "Lithuania"[Mesh]	1844
#170	Search (#167) OR #168	2467
#171	Search (#170) AND #1	4
#172	Select 4 document(s)	4
#173	Search luxembourg*[TIAB]	588
#174	Search "Luxembourg"[Mesh]	464
#175	Search (#173) OR #174	778
#176	Search (#175) AND #1	0
#177	Search europe*[TIAB]	143484
#178	Search "Europe"[Mesh]	989034
#179	Search "European Union"[Mesh]	10045
#180	Search ((#177) OR #178) OR #179	1074955
#181	Search (((((((((("Drug Approval"[Mesh])) OR ("Drug Evaluation"[Mesh])) OR ("Technology Assessment, Biomedical"[Mesh])) OR ("Outcome Assessment (Health Care)"[Mesh])) OR (assessment[TIAB])) OR (approval[TIAB])) OR (evaluation[TIAB])) OR (review[TIAB])) OR ("Advisory Committees"[Mesh])) OR ("Comparative Effectiveness Research"[Mesh])	2265165
#182	Search ((((((pharmaceuticals[TI])) OR (drugs[TI])) OR (medicines[TI])) OR ("Pharmaceutical Preparations"[Mesh]))) AND (((((((reimbursement[TI])) OR (pricing[TI])) OR ("Insurance Coverage"[Mesh])) OR ("Costs and Cost Analysis"[Mesh])) OR ("Insurance, Health, Reimbursement"[Mesh])) OR ("Insurance Benefits"[Mesh]))	6094
#183	Search (#180) AND #182	1335
#184	Search (#183) AND #181	409
#185	Select 409 document(s)	409
