# Supplementary material for: From market access to patient access: overview of evidence-based approaches for the reimbursement and pricing of pharmaceuticals in 36 European countries
Source: Health Res Policy Syst. 2015 Sep 25;13:39. doi: 10.1186/s12961-015-0028-5 (PMC4583728; doi:10.1186/s12961-015-0028-5)
Supplement: Additional file 3: — Sources per country. (DOCX 34 kb) [file 12961_2015_28_MOESM3_ESM.pdf]

Additional file 3: Sources of information per country

**Austria**	
Ministry of Health, Independent Drugs Commission	http://www.bmg.gv.at/home/Schwerpunkte/Medizin/Arzneimittel/Beiraete_und_Kommissionen/Unabhaengige_Heilmittelkommission
Austrian Health Insurance, Drug Evaluation Commission	http://www.sozialversicherung.at/portal27/portal/esvportal/channel_content/cmsWindow?action=2&p_menuid=64715&p_tabid=5
Austrian Health Insurance, Drug Evaluation Commission Members	http://www.sozialversicherung.at/portal27/portal/esvportal/channel_content/cmsWindow?action=2&p_menuid=71895&p_tabid=5
Association of Austrian Social Security Institutions, Code of Procedure for the development of the positive list (Erstattungskodex)	https://www.avsv.at/avi/dokument/binaerdokument_download.pdf?dokid=2004=47&dokStat=0&contTyp=application/pdf
**Belgium**	
National Medical Insurance Association, Pharmaceuticals	http://www.inami.fgov.be/fr/themes/cout-remboursement/par-mutualite/medicament-produits-sante/Pages/default.aspx#.VLAdpns7_-o
National Medical Insurance Association, Costs and Reimbursement	http://www.inami.fgov.be/fr/themes/cout-remboursement/par-mutualite/medicament-produits-sante/remboursement/specialites/procedure/Pages/default.aspx#Peut-on_modifier_les_conditions_de_remboursement_d%e2%80%99une_sp%c3%a9cialit%c3%a9_pharmaceutique?
**Bulgaria**	
National Council on Prices and Reimbursement of Medicinal Products, About the Reimbursement Commission	http://www.ncpr.bg/bg/%D0%BD%D1%81%D1%86%D1%80%D0%BB%D0%BF/%D0%B7%D0%B0-%D1%81%D1%8A%D0%B2%D0%B5%D1%82%D0%B0
Regulations of National Pricing and Reimbursement Commission organization and its administration.	http://www.ncpr.bg/images/demo/Ystroistven_pravilnik_NCPR.pdf
	http://www.ncpr.bg/images/bul_zakonodatelstvo/Naredba-1.pdf
**Cyprus**	
Ministry of Health, Pharmaceutical Services, Committee for human medicines, Website	http://www.moh.gov.cy/moh/phs/phs.nsf/dmlphcomm_gr/dmlphcomm_gr?OpenDocument
Ministry of Health, Committee on the Pricing of Medicines	http://www.moh.gov.cy/moh/phs/phs.nsf/dmlps13_gr/dmlps13_gr?OpenDocument
**Czech Republic**	
State Institute for Drug Control, Regulation of prices and reimbursements for pharmaceuticals	http://www.sukl.eu/medicines/regulation-of-prices-and-reimbursements-for-pharmaceuticals
Law on statutory health insurance, 2011	http://portal.gov.cz/app/zakony/zakonPar.jsp?page=0&idBiblio=75498&recShow=30&nr=376~2F2011&rpp=50#parCnt
Law on statutory health insurance, 1997	http://portal.gov.cz/app/zakony/zakonPar.jsp?page=0&idBiblio=45178&recShow=48&fulltext=&nr=48~2F1997&part=&name=&rpp=100#parCnt
**Germany**	
Federal Joing Committee (G-BA), Composition	https://www.g-ba.de/downloads/17-98-2436/AufbauVorsitz_10-02-2015_deutsch.pdf?
G-BA, Code of Procedure	https://www.g-ba.de/informationen/richtlinien/42/
Institute for Quality and Efficiency in Health Care, General Methods	https://www.iqwig.de/en/methods/methods-papers/general-methods.3020.html

**Denmark**	
Reimbursement Committee, Website	http://laegemiddelstyrelsen.dk/en/topics/statistics,-prices-and-reimbursement/reimbursement/the-reimbursement-committee
**Estonia**	
Estonian Medicines Agency, Reimbursed Pharmaceuticals	http://www.sam.ee/soodusravimid-0
Health Insurance Act, 2002	https://www.riigiteataja.ee/en/eli/527012015004/consolide
Ministry of Social Affairs, Procedure for drafting the positive list,	https://www.riigiteataja.ee/en/eli/509102014007/consolide
**Finland**	
Pharmaceuticals Pricing Board, Website	http://www.stm.fi/en/ministry/boards/pharmaboard/frontpage
Guidelines for preparing an economic evaluation, Ministry of Social Affairs and Health, Pharmaceuticals Pricing Board May 2011	http://www.stm.fi/c/document_library/get_file?folderId=71837&name=DLFE-15657.pdf
**France**	
Haute Autorité de Santé, general methods	http://www.has-sante.fr/portail/upload/docs/application/pdf/general_method_eval_techno.pdf
HAS, Transparency Commission	http://www.has-sante.fr/portail/jcms/c_412210/fr/commission-de-la-transparence
HAS, Methods guide	http://www.has-sante.fr/portail/jcms/c_434715/fr/guide-danalyse-de-la-litterature-et-gradation-des-recommandations?xtmc=&xtcr=1
HAS, submission guidelines	http://www.has-sante.fr/portail/upload/docs/application/pdf/2012-07/notice_depot_ct_v27072012.pdf
Social Security Code	http://legifrance.gouv.fr/affichCode.do;jsessionid=F8C50E8E0082009C193CD2C19D74C3CD.tpdjo16v_2?idArticle=LEGIARTI000006746686&idSectionTA=LEGISCTA000006173275&cidTexte=LEGITEXT000006073189&dateTexte=19991029
**Hungary**	
National Health Insurance Fund Administration, tasks	http://www.oep.hu/felso_menu/rolunk/kozerdeku_adatok/tevekenysegre_mukodesre_vonatkozo_adatok/a_szerv_feladata_alaptevekenysege_es_hatarkore/en_a_szerv_alaptevekenyege_feladata_es_hatarkore
National Institute for Quality and Organisational Development in Healthcare and Medicines (GYEMSZI)	http://www2.gyemszi.hu/site/index.php?page=en/General_information/Deed_of_Foundation_
Technology Appraisal Committee (TAC)	http://fogalomtar.eski.hu/index.php/T%C3%89B
NHIFA legislation monitor	http://www.oep.hu/felso_menu/rolunk/jogszabalyfigyelo/jogszabalyvaltozasok
**Greece**	
Law 4052 on the jurisdiction of the Ministry of Health and Solidarity and Ministry of Employment and Social Protection	http://www.hellenicparliament.gr/Nomothetiko-Ergo/Anazitisi-Nomothetikou-Ergou?law_id=b8755dbe-2947-4716-84f7-dc56a63db441
**Italy**	
AIFA, new regulation on the CTS and CPR (2014)	http://www.agenziafarmaco.gov.it/it/content/regolamento-cts-cpr
AIFA, Commissions	http://www.agenziafarmaco.gov.it/it/content/commissioni-consultive-e-tecnico-scientifiche#cts

**Ireland**	
Health Service Executive, About the Corporate Pharmaceutical Unit	http://www.hse.ie/eng/about/Who/cpu/about.html
HSE, IPHA and APMI agreements	http://www.hse.ie/eng/about/Who/cpu/IPHA_APMI_Agreements.html
HSE, Template for reimbursement application	http://www.hse.ie/eng/about/Who/cpu/Notification_of_Intention_to_Seek_Reimursement_Approval_15.pdf
National Centre for Pharmacoeconomics, pharmacoeconomic evaluations	http://www.ncpe.ie/pharmacoeconomic-evaluations/
NCPE, submission process	http://www.ncpe.ie/submission-process/
**Latvia**	
Cabinet of Ministers Regulations No.899, outpatient reimbursement of drugs and medical devices	http://likumi.lv/doc.php?id=147522
National Health Service, compensations arrangements	http://www.vmnvd.gov.lv/lv/kompensejamie-medikamenti/kompensacijas-kartiba
**Lithuania**	
Ministry of Health Regulation, criteria for inclusion i the positive list	https://www.e-tar.lt/portal/legalAct.html?documentId=TAR.E83CFAB10E7B
Ministry of Health, Reimbursement Commission	http://www.sam.lt/go.php/lit/Ligu-vaistiniu-preparatu-ir-medicinos-pagalbos-priemoniu-kompensavimokomisijos-veikla-
National Health Insurance Fund, reimbursable pharmaceuticals and medical aids	http://www.vlk.lt/sites/en/healthcare-in-lithuania/reimbursable-pharmaceuticals-and-medical-aids
**Luxemburg**	
Ministry of Social Security, organigram	http://www.mss.public.lu/acteurs/index.html
Social Security Medical Control (CMSS)	http://www.mss.public.lu/acteurs/cmss/index.html
Grand Ducal Regulation on reimbursement	http://www.secu.lu/assurance-maladie/reglements/reglement-grand-ducal-modifie-du-12-decembre-2002/
**Malta**	
Directorate of Pharmaceutical Affairs	https://ehealth.gov.mt/HealthPortal/chief_medical_officer/pharm_pol_mon/about_us.aspx
Government Formulary	https://ehealth.gov.mt/HealthPortal/chief_medical_officer/pharm_pol_mon/med_within_ghs/gov_form_list.aspx
Pharmaceutical Health Technology Assessment Unit	https://ehealth.gov.mt/HealthPortal/chief_medical_officer/pharm_pol_mon/pharm_hta_unit.aspx
Government Formulary List Advisory Committee	https://www.gov.mt/en/Government/Government%20of%20Malta/Ministries%20and%20Entities/Officially%20Appointed%20Bodies/Pages/Committees/Government-Formulary-List-Advisory-Committee-.aspx
**Netherlands**	
National Health Care Institut (Zorginstituut Nederlands), Review of Medicines	https://www.zorginstituutnederland.nl/pakket/werkwijze+pakketbeheer/beoordeling+geneesmiddelen
**Poland**	
Ministry of Health, Principles of reimbursement	http://www.mz.gov.pl/en/medication/reimbursement/principles-of-reimbursement
Trensparency Board, Members, Website	http://www.aotm.gov.pl/index.php?id=11
	http://www.aotm.gov.pl/index.php?id=397

**Portugal**	
INFARMED, Economic Review, Price and Reimbursement	http://www.infarmed.pt/portal/page/portal/INFARMED/MEDICAMENTOS_USO_HUMANO/AVALIACAO_ECONOMICA_E_COMPARTICIPACAO
Guidelines for economic drug evaluation studies	http://www.infarmed.pt/portal/page/portal/INFARMED/ENGLISH/PCAEC04_vering.pdf
**Romania**	
National Agency for Medicines and Medical Devices, Health Technology Assessment Unit	http://www.anm.ro/anmdm/en/evaluare_tehnologii_medicale.html
Ministry of Public Health, organigram	http://www.ms.ro/upload/Anexa%201%20organigrama%2015.04.2014%20-%202.pdf
AMMD, relevant regulation	http://www.anm.ro/anmdm/en/med_legislatie.html
**Slovenia**	
Health Insurance Institute of Slovenia, rules on the classification of medicines on the positive list	http://www.zzzs.si/zzzs/info/egradiva.nsf/o/54B4834F0D9B092DC1256CB40045469F?OpenDocument
Appointment of Commission members	http://www.zzzs.si/zzzs/info/egradiva.nsf/o/42499159924406F2C12573C900342B88
**Slovakia**	
Ministry of Health, Categorization of medicines	http://www.health.gov.sk/?kategorizacia-liekov
MoH, Guide for pharmacoeconomic analyses	http://www.health.gov.sk/?Dokumenty-Farmako-ekonomicky-a-medicinsko-ekonomicky-rozbor
MoH, Regulation no. 07045-41 / 2012-OL, Scope of Price regulation	http://www.health.gov.sk/Clanok?Opatrenie-07045-41
MoH, Establishing the positive list	http://www.health.gov.sk/Clanok?oprava-v-opatreni-zo-16-3-2005-ktorym-sa-meni-a-doplna-opatrenie-ktorym-sa-vydava-zoznam-lieciv-a-liekov-uhradzanych-na-zaklade-zp
**Spain**	
Directorate General for Basic Services Portfolio and Pharmacy, information	http://www.msssi.gob.es/organizacion/ministerio/organizacion/sgralsanidad/dgcbssnsyfF.htm
General Health Law	http://www.msssi.gob.es/organizacion/sns/planCalidadSNS/pdf/transparencia/Ley_14_86_GRAL_SANIDAD_1.pdf
Law 16/2003 on the cohesión and quality of the National Health Service	http://www.msssi.gob.es/organizacion/sns/planCalidadSNS/pdf/transparencia/LEY_COHESION_Y_CALIDAD.pdf
Royal Decree 200/2012 on the basic organizational structure of the Ministry of Health	http://www.boe.es/diario_boe/txt.php?id=BOE-A-2012-1034
**Sweden**	
TLV, Pricing and reimbursement of medicines	http://www.tlv.se/In-English/medicines-new/pricing-and-reimbursement-of-medicines/
TLV, Submission guidelines	http://www.tlv.se/In-English/medicines-new/apply-for-a-price-or-reimbursement/
TLV, Pharmaceutical reviews	http://www.tlv.se/Upload/English/ENG-handbook.pdf
**UK - Scotland**	
Scottish Medicines Consortium, Remit	https://www.scottishmedicines.org.uk/About_SMC/What_we_do/Remit
SMC, New Drugs Committe Members	https://www.scottishmedicines.org.uk/About_SMC/Who_we_are/Membership/NDC_Membership

The Scottish Government, Guidance to further Strengthen the safe and effective use of new medicines across the NHS	http://www.sehd.scot.nhs.uk/cmo/CMO%282012%2901.pdf
SMC, Submission guidance	https://www.scottishmedicines.org.uk/Submission_Process/Submission_Process
SMC, Guidance process	https://www.scottishmedicines.org.uk/files/sign/SIGN_SMC__Algorithm_MAR10_new.pdf
**UK - England**	
National Institute for Health and Care Excellence, guide to the process of technology appraisal	https://www.nice.org.uk/article/pmg19/chapter/Acknowledgements
NICE, guide to the methods of technology appraisal 2013	https://www.nice.org.uk/article/pmg9/chapter/Foreword
NICE, specification for company submission of evidence	https://www.nice.org.uk/about/what-we-do/our-programmes/nice-guidance/nice-technology-appraisal-guidance
**Croatia**	
Ministry of Health, specifications for price-setting	http://narodne-novine.nn.hr/clanci/sluzbeni/2013_07_83_1801.html
Ministry of Health, specifications for reimbursement	http://narodne-novine.nn.hr/clanci/sluzbeni/2013_07_83_1803.html
**Iceland**	
Icelandic Medicine Pricing and Reimbursement Committee, details in English	http://www.lgn.is/index.php?pageid=62
**Macedonia**	
Ministry of Health, Amendment and supplement to the Health Insurance Act from 17.02.2012 (Official Gazette 26/2012), Paragraph 9	http://www.fzo.org.mk/WBStorage/Files/3.%20ZAKON%20ZA%20IZMENUVANJE%20I%20DOPOLNUVANJE%20NA%20ZAKONOT%20ZA%20ZDRAVSTVENOTO%20OSIGURUVANJE%2026%20od%202012.pdf
Decree on the method and methodology on determining and adoption of pharmaceutical reimbursement list covered by the Health Insurance Fund of Macedonia (Official Gazette 116/2012)	http://www.fzo.org.mk/WBStorage/Files/Lista%20na%20Lekovi%20Predlog.pdf
**Montenegro**	
Ministry of Health, Regulation on the basic and supplementary lists of medicines	http://www.fzocg.me/docs/166/uredbom_o_kriterijumima_za_utvrdjivanje_osnovne_i_dopunske_liste_ljekova.pdf
MoH, Decision on the List of medicines prescribed and issued at the expense of the Health Insurance Fund	http://www.sluzbenilist.me/PravniAktDetalji.aspx?tag=%7BEC557B08-3AC7-404F-8A4D-9443C573FAC3%7D
**Turkey**	
Social Security Institution, Process and Principles of the Reimbursement Commission	http://www.sgk.gov.tr/wps/wcm/connect/ab787daf-84f3-4401-9f1a-d5fea06660ec/yonerge_03072014.pdf?MOD=AJPERES&CACHEID=ab787daf-84f3-4401-9f1a-d5fea06660ec
Social Security Institution, Vision, Mission and Core Values	http://www.sgk.gov.tr/wps/portal/en

**Serbia**	
Ministry of Health, Regulation on the conditions, criteria, methods and procedures for placing the medicinal product on the positive list	http://www.rfzo.rs/download/pravilnici/lekovi/Pravilnik_o_kriterijumima10%20122014.pdf
**Liechtenstein**	
Liechtenstein Official Gazette, Ordinance on the Social Security Law	https://www.gesetze.li/DisplayLGBl.jsp?Jahr=2000&Nr=74
**Norway**	
Norwegian Medicines Agency, Pricing and Reimbursement	http://www.legemiddelverket.no/English/price_and_reimbursement/Sider/default.aspx
NoMA, General Reimbursement	http://www.legemiddelverket.no/English/price_and_reimbursement/general_reimbursement/Sider/default.aspx
NoMA, application for reimbursement	http://www.legemiddelverket.no/English/price_and_reimbursement/application_for_reimursement/Sider/default.aspx
NoMa, Guidelines on how to conduct pharmacoeconomic analyses	http://www.legemiddelverket.no/English/price_and_reimbursement/application_for_reimursement/Lists/PageAttachments/default/NO/Pharmacoeconomic%20guidelines%20-%20Norway.pdf
**Switzerland**	
Federal Office of Public Health, Composition of the Federal Drug Commission	http://www.admin.ch/ch/d/sr/832_102/a37e.html
Federal Office of Publc Health, Handbook on the positive list	http://www.bag.admin.ch/themen/krankenversicherung/06492/07568/index.html
