# Supplementary material for: From market access to patient access: overview of evidence-based approaches for the reimbursement and pricing of pharmaceuticals in 36 European countries
Source: Health Res Policy Syst. 2015 Sep 25;13:39. doi: 10.1186/s12961-015-0028-5 (PMC4583728; doi:10.1186/s12961-015-0028-5)
Supplement: Additional file 4: Table S1. — Overview of evidence-based decision-making processes for pharmaceutical coverage in 36 countries. (DOCX 62 kb) [file 12961_2015_28_MOESM4_ESM.pdf]

Table 1 Overview of evidence-based decision-making processes for pharmaceutical coverage in 36 countries

**Country**	**Institution in charge of coverage**	**Remit**	**Purpose of appraising value**	**Initial evidence report**		**Evidence assessment team**	**Appraisal Committee (members, term)**	**Reassessment**			**Restrictions**	**Final political decision**
				**Producer**	**Costs considered**			**Regular**	**Ad hoc**			
									**Initiator**	**Reason**		
Austria	HVB	NAM, POM<br>OP	RS<br>maximum price	M	CbC	HEK at HVB	HEK (20/5)	no	HEK, M	E,I	I, MB,PrG	HVB
Belgium	INAMI	NAM, RM<br>OP, IP	RS (level)	M	CbC	INAMI staff	CRM at INAMI (31/6)	innov. drugs 1.5-3y	CRM/ MoSA	-	I, PaG, PS	MoSA
**Bulgaria**	CPR (MoH)	NAM, POM<br>OP,IP	RS<br>maximum price, PR classifi-cation	M	CbC	CPR	CPR (7/-)	no	CPR/M	E	I, DD, PaG	CPR
**Croatia**	HZZO	NAM<br>OP, IP	RS (level) PR classifi-cation	M	CbC	RC at HZZO	RC (13/-)	no	M	I	PaG, PS, PrG	HZZO Board
Cyprus	CHM (MoH)	NAM<br>OP, IP	RS<br>tenders	M	yes	CPD at CHM	CHM (12/3)	-	-	-	PaG	CHM

Legend: CbC = case by case; DD = duration of disease; E = evidence; I = indication; IP = inpatient; M = manufacturer; MB = Medical Benefit; NAM = newly authorized medicines; OP = outpatient; PaG = patient group; POM = prescription-only medicine; PR = price referencing; PrG = professional group; PS = product-specific; RM = reimbursed medicines; RS = reimbursement status; TP = treatment programme; y = years; "-" = no information found. **Institutional abbreviations:** CHM - Committee for Human Medicines; CPD - Clinical Pharmacy Department; CPR - Commission for Pricing and Reimbursement; CRM - Drug Reimbursement Committee; HEK – Drugs Evaluation Commission; HVB - Association of Austrian Social Security Institutions; HZZO - Croatian Institute for Health Insurance; INAMI - Belgian Statutory National Medical Insurance Association; MoH – Ministry of Health; MoSA - Ministry of Social Affairs; RC – Reimbursement Committee.

Table 1 Overview of evidence-based decision-making processes for pharmaceutical coverage in 36 countries (cont.)

Country	Institution in charge of coverage	Remit	Purpose of appraising value	Initial evidence report		Evidence assessment team	Appraisal Committee (members, term)	Reassessment			Restrictions	Final political decision
				Producer	Costs considered			Regular	Ad hoc			
									Initiator	Reason		
**Czech Republic**	SÚKL	IP - NAM, POM<br>OP – all reimburs.	RS maximum price	M	CbC	CAU at SÚKL	CAU (28/-)	3y	-	-	I, PaG, PrG	SÚKL
Denmark	DHMA	NAM, RM, OP	RS	M	CbC	RC at DHMA or DACEHTA	RC (7/4)	no	RC	-	I, DD, PaG	DHMA
**Estonia**	PC (MoSA)	NAM, RM<br>OP	RS (level)<br>maximum price	M	yes	PC (EHIF/ SAM members)	PC (8/-)	no	M, EHIF, patients	I, PC	I, PaG	MoSA
Finland	PPB (MoSA)	NAM, RM<br>OP	RS<br>maximum price	M	yes	PPB & KELA	PPB (7/3)	5y (3y new active subst.)	-	-	I, DD, PaG	PPB
France	HAS	NAM, POM<br>OP, IP	RS (level)<br>price negotiation	M or MoHSS	CbC	HAS staff	CT at HAS (26/3)	5y	MoHSS	I	I, MB	MoHSS
Germany	G-BA	NAM, POM<br>OP, IP	RS<br>price negotiation	M	CbC	IQWiG and/or G-BA	G-BA (13/4)	no	G-BA, M	-	PaG, quality	G-BA

Legend: CbC = case by case; DD = duration of disease; I = indication; IP = inpatient; M = manufacturer; MB = Medical Benefit; NAM = newly authorized medicines; OP = outpatient; PaG = patient group; PC = price change; POM = prescription-only medicine; PrG = professional group; RM = reimbursed medicines; RS = reimbursement status; y = years; “-”= no information found.
Institutional abbreviations: CAU - Price and Reimbursement Regulation; CT - Transparency Committee; DACEHTA - Danish Centre for Health Technology Assessment; DHMA - Danish Health and Medicines Authority; EHIF - Estonian Health Insurance Fund; G-BA - Federal Joint Committee; HAS - French National Authority for Health; IQWiG - Institute for Quality and Efficiency in Health Care; KELA - Social Insurance Institution; MoHSS – Ministry of Health and Social Security; MoSA - Ministry of Social Affairs; PC –Pharmaceuticals Committee; PPB - Pharmaceuticals Pricing Board; RC –Reimbursement Committee; SAM –State Agency of Medicines; SÚKL - State Institute for Drug Control.

Table 1 Overview of evidence-based decision-making processes for pharmaceutical coverage in 36 countries (cont.)

Country	Institution in charge of coverage	Remit	Purpose of appraising value	Initial evidence report		Evidence assess-ment team	Appraisal Committee (members, term)	Reassessment			Restrictions	Final political decision
				Producer	Costs considered			Regular	Ad hoc			
									Initiator	Reason		
Greece	MoH/EOF	NAM, POM<br>OP	RS (level)<br>PR classification	none	-	none	EOF	-	-	-	-	MoH
**Hungary**	NHIFA	NAM, POM<br>OP, IP	RS<br>maximum price	M	yes	GYEMSZI	TAC at NHIFA (8/-)	no	M	PC	I, PaG	NHIFA
**Iceland**	MoW	NAM, RM, POM<br>OP	RS<br>maximum price	M	yes	LGN at MoW	LGN (5+/4)	-	-	-	I, PaG	LGN at MoW
Ireland	HSE	NAM<br>OP, IP	RS (level)	M	yes	NCPE	CPU at HSE (-/-)	-	-	-	I, DD	CPU at HSE
Italy	AIFA	NAM<br>OP, IP	RS<br>maximum price	M	CbC	CTS at AIFA	CPR at AIFA (10/-)	-	AIFA, M	I, PC	I, DD, PS, PaG, PrG	AIFA

Legend: CbC = case by case; DD = duration of disease; I = indication; IP = inpatient; M = manufacturer; NAM = newly authorized medicines; OP = outpatient; PaG = patient group; PC = price change; POM = prescription-only medicine; PR = price referencing; PrG = professional group; PS = product-specific; RM = reimbursed medicines; RS = reimbursement status; TP = treatment programme; "-" = no information found. **Institutional abbreviations:** AIFA - Italian Medicines Agency; CPR - Pricing and Reimbursement Committee; CPU - Corporate Pharmaceutical Unit; CTS - Technical and Scientific Commission; EOF – National Organization for Medicines; GYEMSZI - National Institute for Quality- and Organizational Development in Healthcare and Medicines; HSE - Health Service Executive; LGN - Icelandic Medicine Pricing and Reimbursement Committee; MoH – Ministry of Health; MoW - Ministry of Welfare; NCPE - National Centre for Pharmaco-economics; NHIFA - National Health Insurance Fund Administration; TAC - Technology Appraisal Committee.

Table 1 Overview of evidence-based decision-making processes for pharmaceutical coverage in 36 countries (cont.)

Country	Institution in charge of coverage	Remit	Purpose of appraising value	Initial evidence report		Evidence assessment team	Appraisal Committee (members, term)	Reassessment			Restrictions	Final political decision
				Producer	Costs considered			Regular	Ad hoc			
									Initiator	Reason		
**Latvia**	NHS	NAM<br>OP, IP	RS (level)<br>maximum price	M	yes	NHS staff	RMMED at NHS (-/-)	2y (appr.)	NHS, M	PC	I	NHS
Liechten-stein	HSC (adopts Swiss decisions)	see Switzerland										Government
**Lithuania**	SAM (MoH)	NAM, POM<br>OP	RS maximum price	M	yes	PhD at MoH	SAM (14/-)	5y	-	-	I, DD, PaG, PrG	MoH
Luxembourg	MoH	NAM OP, IP	RS (level)<br>maximum price	-	yes	CMSS at MoH	CMSS at MoH	-	-	-	prescription-based	MoH
**Macedonia**	MoH	NAM, POM<br>OP,IP	RS	M	yes	ad hoc groups at MoH	ad hoc expert commissions at MoH (17/1)	no	M, MoH HIF	E, I, PC	I, DD, PrG, PS	MoH

Legend: DD = duration of disease; I = indication; IP = inpatient; M = manufacturer; NAM = newly authorized medicines; OP = outpatient; PaG = patient group; PC = price change; POM = prescription-only medicine; PrG = professional group; PS = product-specific; RS = reimbursement status; TP = treatment programme; y = years; "-"= no information found. **Institutional abbreviations:** CMSS – Pharmaceutical Reimbursement Commission; HIF – Health Insurance Fund; HSC – Health Services Commission; MoH - Ministry of Health; NHS - National Health Service; PhD - Department of Pharmacy; RMMED - Reimbursable medicines and medical equipment division; SAM –Positive Drug List Committee.

Table 1 Overview of evidence-based decision-making processes for pharmaceutical coverage in 36 countries (cont.)

Country	Institution in charge of coverage	Remit	Purpose of appraising value	Initial evidence report		Evidence assessment team	Appraisal Committee (members, term)	Reassessment			Restrictions	Final political decision
				Producer	Costs considered			Regular	Ad hoc			
									Initiator	Reason		
**Malta**	GFLAC	NAM, RM<br>OP,IP	RS<br>maximum price	M	CbC	DPA at MoH	GFLAC (9/2)	no	M	-	PrG	SPH
**Montenegro**	FZZO	NAM, RM, POM<br>OP, IP	RS	M	CbC	PC at FZZO	FZZO Board (13/4)	no	MoH, M	I	-	Board of FZZO
The Netherlands	ZIN	NAM<br>OP, IP (high cost)	RS (level)	M	CbC	ZIN staff	ZIN Board (3/8)	4y	M, OS	I	I, MB, PaG	MoHWS
Norway	NoMA	NAM, POM<br>OP	RS	M	yes	DoP at NoMA	NoMA (unclear)	-	-	-	DD	NoMA
**Poland**	MoH	NAM<br>OP, IP	RS	M	CbC	AOTM	MoH (-/-)	-	M, OS	-	I, PaG	MoH
Portugal	INFARMED	NAM<br>OP, IP	RS	M	CbC	INFARMED staff	INFARMED Committee (45/3)	3y	-	-	PS, PaG	MoH (OP)

Legend: CbC = case by case; DD = duration of disease; I = indication; IP = inpatient; M = manufacturer; NAM = newly authorized medicines; OP = outpatient; OS= other stakeholders; PaG = patient group; PC = price change; POM = prescription-only medicine; PrG = professional group; RM = reimbursed medicines; RS = reimbursement status; "-"= no information found. **Institutional abbreviations:** AOTM - Agency for Health Technology Assessment in Poland; CFH - Commission for pharmaceutical help; DoP – Department of Pharmacoeconomics; DPA - Directorate of Pharmaceutical Affairs; FZZO – Health Insurance Fund; GFLAC - Government Formulary List Advisory Committee; INFARMED - National Authority of Medicines and Health Products; MoH - Ministry of Health; MoHWS - Ministry of Health, Welfare and Sport; NoMA – Norwegian Medicines Agency; PC –Pharmaceutical Commission; SPH - Superintendent for Public Health; CVZ - Medicinal Products Reimbursement Committee of the Dutch Healthcare Insurance Board; ZIN – National Health Care Institute.

Table 1 Overview of evidence-based decision-making processes for pharmaceutical coverage in 36 countries (cont.)

Country	Institution in charge of coverage	Remit	Purpose of appraising value	Initial evidence report		Evidence assess-ment team	Appraisal Committee (members, term)	Reassessment			Restrictions	Final political decision
				Producer	Costs considered			Regular	Ad hoc			
									Initiator	Reason		
**Romania**	MoPH	NAM, RM<br>OP	RS (level)	M	CbC	AMMD	TC at MoPH (4/-)	quarterly (prices)	-	-	I, PS, PrG, DD	MoPH
**Serbia**	RFZO	NAM, RM, POM<br>OP,IP	RS maximum price	M	yes	RFZO staff	CSK at RFZO (7/-)	no	MoH, CSK	I, PC	I, PaG, PS, PrG	MoH
**Slovakia**	MoH	NAM, POM<br>OP	RS	M & MoH staff	yes	CCM at MoH	CCM (10/-)	quarterly (RS)	-	-	I, DD	MoH
**Slovenia**	ZZZS	NAM, POM<br>OP, IP (high cost)	RS (level) price negotiation	M, ZZZS	yes	DC at ZZZS	DC (16/4)	no	ZZZS, M	E, I	I, DD, PaG, PrG	ZZZS
**Spain**	IPC (pricing) & DGCF (reimburse-ment)	NAM, RM<br>OP, IP	RS<br>maximum price	M & DGCF	yes	DGCF/IPC	DGCF/IPC (-/-)	max. 3y	-	-	PS	DGCF

Legend: CbC = case by case; DD = duration of disease; E = evidence; I = indication; IP = inpatient; M = manufacturer; NAM = newly authorized medicines; OP = outpatient; PaG = patient group; PC = price change; POM = prescription-only medicine; PrG = professional group; PS = product-specific; RM = reimbursed medicines; RS = reimbursement status; y = years; “-”= no information found. **Institutional abbreviations:** AMMD – Romanian National Agency for Medicines and Medical Devices; CCM - Categorisation Commission for Medicines; CSK – Central Expert Committee for Medicines; DC - Drugs Committee; DGCF - Directorate General for Basic Services Portfolio and Pharmacy; IPC - Inter-Ministerial Pricing Commission; MoH - Ministry of Health; MoPH - Ministry of Public Health; RFZO – Republic Fund for Health insurance; TC – Transparency Committee; ZZZS - Health Insurance Institute of Slovenia

Table 1 Overview of evidence-based decision-making processes for pharmaceutical coverage in 36 countries (cont.)

**Country**	**Institution in charge of coverage**	**Remit**	**Purpose of appraising value**	**Initial evidence report**		**Evidence assessment team**	**Appraisal Committee (members, term)**	**Reassessment**			**Restrictions**	**Final political decision**
				**Producer**	**Costs considered**			**Regular**	**Ad hoc**			
									**Initiator**	**Reason**		
Sweden	TLV	NAM, RM, POM<br>OP	RS<br>maximum price	M & TLV	yes	PBB at TLV	PBB (7/-)	Yes (variable)	TLV, M	-	PS, PaG	PPB at TLV
Switzerland	BAG	NAM, RM<br>OP, IP	RS<br>maximum price	M	yes	EAK at BAG	EAK (16/-)	-	-	-	I, supply	BAG
**Turkey**	SGK	NAM<br>OP, IP	RS (level)<br>PR classification<br>maximum price	M	-	MEAC* at SGK	ÖK* at SGK (10/-)	-	-	-	-	ÖK*
UK - England & Wales	NICE	NAM, RM OP, IP	RS	M & NICE/others	yes	NICE staff and/or external centres	AC at NICE (var.)	1-3y	-	-	I, PaG, PrG, TP	NHS
UK - Scotland	SMC	NAM, POM<br>OP, IP	RS	M	yes	SMC staff	NDC at SMC	no	AD&TC	E	I, PaG, PrG, TP	AD&TC (NHSS)

Legend: E = evidence; I = indication; IP = inpatient; M = manufacturer; NAM = newly authorized medicines; OP = outpatient; OS= other stakeholders; PaG = patient group; POM = prescription-only medicine; PR = price referencing; PrG = professional group; PS = product-specific; RM = reimbursed medicines; RS = reimbursement status; TP = treatment programme; y = years; "-" = no information found. **Institutional abbreviations:** AC - Appraisal Committee; AD&TC - Area Drugs and Therapeutic Committee; BAG – Federal Office of Public Health; EAK – Federal Drug Commission; MEAC – Medical and Economic Evaluation Commission; NHS - National Health Service; NHSS - National Health Service Scotland; NICE - National Institute for Health and Care Excellence; SMC - Scottish Medicines Consortium; NDC - New Drugs Committee (Scotland); ÖK – Reimbursement Commission; PBB - Pharmaceutical Benefits Board; SGK – Social Security Institution; TLV - Dental and Pharmaceutical Benefits Agency // *MEAC and ÖK comprise members from the Social Security Institution, the General Directorate of Pharmaceuticals and Pharmacy and the Ministry of Finance.
