# Supplementary material for: From market access to patient access: overview of evidence-based approaches for the reimbursement and pricing of pharmaceuticals in 36 European countries
Source: Health Res Policy Syst. 2015 Sep 25;13:39. doi: 10.1186/s12961-015-0028-5 (PMC4583728; doi:10.1186/s12961-015-0028-5)
Supplement: Additional file 1: — Overview of comparative peer-reviewed publications on elements of reimbursement and pricing. (PDF 29 kb) [file 12961_2015_28_MOESM1_ESM.pdf]

Additional document 1 Overview of comparative peer-reviewed publications on elements of pricing and reimbursement since 2010
(this is an indicative overview of publications focusing on institutional practices related to the process of reimbursement and/or pricing, and examining a minimum of three countries ncluding at least one European country)
Full references are provided on page 2.

					European Countries																																								
					EU28																												Associated & Candidate					Additional OECD Countries							
**Authors**	**Year**	**Focus**	**Number of countries**	**Data collection**	AT	BE	BG	CY	CZ	DE	DK	EE	EL	ES	FI	FR	GB	HR	HU	IE	IT	LT	LU	LV	MT	NL	PL	PT	RO	SE	SI	SK	CH	IS	LI	NO	TR	AU	CA	IL	JP	KR	MX	NZ	US
Barnieh et al. (1)	2014	procedural elements, outpatient perscription drugs	34	2011													E S																												
Allen et al. (2)	2013	construction of archetypes for the role of national HTA systems in decision-making	33	unclear													E S																												
Leopold et al. (3)	2013	pricing and reimbursement challenges in personalised medicine	29	2011													E																												
Riedel et al. (4)	2013	economic evaluation (type, perspective, utility values, discounting, modeling)	12	2011													E																												
Spinner et al. (5)	2013	impact of clinical evidence base on decision-making (comparators, selective decision-making, indirect comparisons etc.)	3	2011													E																												
Cleemput et al. (6)	2012	"accountability for reasonableness" conditions (transparency, relevance, revisability, enforcement)	5	2012																																									
Franken et al. (7)	2012	objective, Implementation modalities and accountability	5	2012																																									
Kleijnen et al. (8)	2012	methodological elements in comparative effectiveness research	29	2011													E S																												
Leopold et al. (9)	2012	external reference pricing (basket composition, price calculation)	28	2009-2010													*																												
Vogler (10)	2012	generics policies (price control, price linkage, substitution, INN prescribing)	29	2011													*																												
Mauskopf et al. (11)	2011	submission requirements (systematic review/meta-analysis, critical appraisal etc.)	10	unclear													E S																												
Stafinski et al. (12)	2011	procedural elements in reimbursement decisions on new technologies	21	2010													*																												
Vogler et al. (13)	2011	pricing and reimbursement elements based on Pharmaceutical Health Information System indicators	28	2010													*																												
Drummond et al. (14)	2010	case studies focusing on price and reimbursement status differences	4	2009													E S																												
Levy et al. (15)	2010	process for inclusion in national reimbursement list (formulary)	5	2009													S																												

*(Note: E = England; S = Scotland; *no (clear) distinction within Great Britain)*

1. Barnieh L, Clement F, Harris A, Blom M, Donaldson C, Klarenbach S, Husereau D, Lorenzetti D, Manns B: A systematic review of cost-sharing strategies used within publicly-funded drug plans in member countries of the organisation for economic co-operation and development. PLoS One 2014, 9:e90434.

2. Allen N, Pichler F, Wang T, Patel S, Salek S: Development of archetypes for non-ranking classification and comparison of European National Health Technology Assessment systems. Health Policy 2013, 113:305-312.

3. Leopold C, Vogler S, Habl C, Mantel-Teeuwisse AK, Espin J: Personalised medicine as a challenge for public pricing and reimbursement authorities - A survey among 27 European countries on the example of trastuzumab. Health Policy 2013, 113:313-322.

4. Riedel R, Repschlager U, Griebenow R, Breitkopf S, Schmidt S, Guhl A: International standards for health economic evaluation with a focus on the German approach. J Clin Pharm Ther 2013, 38:277-285.

5. Spinner DS, Birt J, Walter JW, Bowman L, Mauskopf J, Drummond MF, Copley-Merriman C: Do different clinical evidence bases lead to discordant health-technology assessment decisions? An in-depth case series across three jurisdictions. ClinicoEconomics and outcomes research : CEOR 2013, 5:69-85.

6. Cleemput I, Franken M, Koopmanschap M, le Polain M: European drug reimbursement systems' legitimacy: five-country comparison and policy tool. Int J Technol Assess Health Care 2012, 28:358-366.

7. Franken M, le Polain M, Cleemput I, Koopmanschap M: Similarities and differences between five European drug reimbursement systems. Int J Technol Assess Health Care 2012, 28:349-357.

8. Kleijnen S, George E, Goulden S, d'Andon A, Vitre P, Osinska B, Rdzany R, Thirstrup S, Corbacho B, Nagy BZ, et al: Relative effectiveness assessment of pharmaceuticals: similarities and differences in 29 jurisdictions. Value Health 2012, 15:954-960.

9. Leopold C, Vogler S, Mantel-Teeuwisse AK, de Joncheere K, Leufkens HG, Laing R: Differences in external price referencing in Europe: a descriptive overview. Health Policy 2012, 104:50-60.

10. Vogler S: The impact of pharmaceutical pricing and reimbursement policies on generics uptake: implementation of policy options on generics in 29 European countries - an overview. Generics and Biosimilars Initiative Journal 2012, 1:93-100.

11. Mauskopf J, Walter J, Birt J, Bowman L, Copley-Merriman C, Drummond M: Differences among formulary submission guidelines: implications for health technology assessment. Int J Technol Assess Health Care 2011, 27:261-270.

12. Stafinski T, Menon D, Davis C, McCabe C: Role of centralized review processes for making reimbursement decisions on new health technologies in Europe. ClinicoEconomics and Outcomes Research (Dovepress) 2011, 3:117-186.

13. Vogler S, Habl C, Bogut M, Voncina L: Comparing pharmaceutical pricing and reimbursement policies in Croatia to the European Union Member States. Croat Med J 2011, 52:183-197.

14. Drummond M, Jönsson B, Rutten F, Stargardt T: Reimbursement of pharmaceuticals: reference pricing versus health technology assessment. The European journal of health economics : HEPAC : health economics in prevention and care 2011, 12:263-271.

15. Levy AR, Mitton C, Johnston KM, Harrigan B, Briggs AH: International comparison of comparative effectiveness research in five jurisdictions: insights for the US. Pharmacoeconomics 2010, 28:813-830.
